# A novel integrated extraction protocol for multi-omic studies in heavily degraded samples

**DOI:** 10.1038/s41598-024-67104-8

**Published:** 2024-07-30

**Authors:** Byron Boggi, Jack D. A. Sharpen, George Taylor, Konstantina Drosou

**Affiliations:** 1 Faculty of Biology, Medicine and Health, Division of Cell Matrix Biology and Regenerative Medicine, University of Manchester, Manchester, M13 9PL UK; 2 Faculty of Biology, Medicine and Health, Research and Innovation, University of Manchester, Manchester, M13 9PG UK; 3 Manchester Institute of Biotechnology, University of Manchester, Manchester, M1 7DN UK

**Keywords:** Degraded DNA, Ancient DNA (aDNA), Integrated extraction protocol, Multi-omic protocol, Evolutionary biology, Biological techniques, Genetics, Environmental sciences

## Abstract

The combination of multi-omic techniques, such as genomics, transcriptomics, proteomics, metabolomics and epigenomics, has revolutionised studies in medical research. These techniques are employed to support biomarker discovery, better understand molecular pathways and identify novel drug targets. Despite concerted efforts in integrating omic datasets, there is an absence of protocols that integrate all four biomolecules in a single extraction process. Here, we demonstrate for the first time a minimally destructive integrated protocol for the simultaneous extraction of artificially degraded DNA, proteins, lipids and metabolites from pig brain samples. We used an MTBE-based approach to separate lipids and metabolites, followed by subsequent isolation of DNA and proteins. We have validated this protocol against standalone extraction protocols and show comparable or higher yields of all four biomolecules. This integrated protocol is key to facilitating the preservation of irreplaceable samples while promoting downstream analyses and successful data integration by removing bias from univariate dataset noise and varied distribution characteristics.

Over the last decade, multi-omic techniques have been in the forefront of biological sciences. The analytical power integrating multiple datasets has facilitated major advances in medicine, microbiology, modern human disease studies^1,2^ ecology and agriculture^3^. The purpose of multi-omic studies is to provide a deeper understanding of the interplay between multiple layers of biological regulation and in particular to support biomarker discovery, predict novel drug targets, increase diagnostic power for health and improve disease prognosis^4^. Despite the potential of machine learning technologies, multi-omic data integration faces many challenges such as data normalisation and compatibility, clustering, functional characterisation and visualisation which confound their analyses^5,6^. An added level of complexity is that multi-omics datasets are derived from blending data from individual workflows, different processing platforms, and intra-sample biomolecule preservation.

In heavily degraded remains, such as ancient, forensic, archival or even clinical Formalin-Fixed-Paraffin-Embedded (FFPE) samples, the situation is even more challenging. Sample heterogeneity, limited DNA quantity, fragmentation, degradation, contamination, post-mortem damage and chemical modifications can all pose challenges and introduce further bias in downstream analyses which complicates data integration^7,8^. For these reasons, multi-omic analyses and data integration in the ancient DNA (aDNA) field have been massively overlooked. To this day most research focusses either on genomics, or proteomics, and most often individually, rather than through a combined approach. A major drawback is that archaeological samples are rare and irreplaceable so there is a need to increase the amount of data that can be derived from a single biopsy. Integration on the workflow level, i.e., the extraction of two or more biomolecules from a single sample/biopsy, has been limited. The few examples where it has been attempted in the literature have largely concentrated on the combination of DNA and protein, for example from dental remains^9^ and dental calculus in particular^10^. Furthermore, in the former case, the efficacy of the combined protocol was not compared to similar, individual protocols, making it difficult to assess the effectiveness of the method. The latter study reported a nearly 50% decrease in endogenous DNA recovered when compared to a DNA-only protocol, likely due to the method requiring long incubation times (72 h) with no nuclease protection. Previous studies have shown that an optimised silica-based spin column can effectively co-extract DNA, RNA and protein^11^ and commercial kits have been available for a number of years (AllPrep DNA/RNA/Protein Mini Kit—Qiagen). Such kits could likely be optimised for aDNA and peptide recovery in a similar fashion to other proprietary chemistry^12^. One of the main practical challenges in co-extracting aDNA and peptides is the long incubation time during extraction and fragile nature of aDNA, whereby inhibition of nucleases is essential to preserve as much genomic content as possible. As with most DNA extraction protocols, proteinases are the gold standard. However, such treatments are not amenable to proteomic workflows for already degraded peptides in bottom-up workflows. Hence, inactivation of nucleases could be achieved with a strong denaturant, such as methanol, where loss of structure will effectively inhibit nuclease activity. Furthermore, such denaturants will lead to the precipitation of genomic and proteomic contents of the extracted sample, partially purify the sample and facilitate the recovery of polar and non-polar small molecules. As such, solvent mixtures (chlorofolm:methanol)^13,14^, have been adopted as the gold standard for lipids and metabolites for over 70 years and are widely used for the co-extraction of protein^15^.

Another popular biphasic organic extraction protocol for lipids and metabolites, adopts Methyl-tert-butyl-ether (MTBE) as a safer alternative to chloroform and allows faster and cleaner recovery of a broad range of lipid classes^16^. As with chloroform:methanol this protocol has been shown to effectively co-extract proteomic content and provide a comprehensive view of cell signalling mechanisms^17^.

However, a protocol that includes DNA, lipids, proteins, and metabolites does not currently exist in published literature for ancient, forensic or clinical samples. Here we demonstrate a novel, fully integrated extraction protocol implemented on artificially degraded porcine brains that is suitable for degraded samples. We designed our protocol the following rationale:

First, we consider that biomolecular analyses of archaeological remains involve destructive sampling and require a significant amount of initial template for separate extraction methods. In addition, such analyses are often complemented by radiocarbon dating and stable isotope analysis, which require additional sample material. Ancient soft tissues, such as hair, skin, muscle and brain, have rarely been considered for biomolecular and biomedical research because these tissues are often part of an intact, mummified individual, and are subject to different sampling considerations than skeletal remains. Therefore, by combining the extraction of aDNA, proteins, and lipids from a single biopsy, we significantly reduce the amount of template material required, thus preserving a finite and irreplaceable resource, and facilitating greater depth of analysis that would otherwise be prohibited due to the requirement for destructive sampling.

Second, of all the soft tissues preserved in the archaeological record, brain is one of the most overlooked tissues as its recovery was previously considered to be rare phenomenon. This tendency to refer to it as rare has caused a paucity of studies into ancient brain tissue analyses, as well as studies in brain preservation and degradation mechanisms^18^. However, to date there are over 200 independent published reports about preserved brain specimens from a wide range of depositional environments, and around a thousand more reports about brain specimens dating back to the seventeenth century, in which the brain appears in various states of preservation^19–24^. Moreover, forensic studies in brain tissue have found that DNA is relatively resistant to putrefaction and in contrast to bone, the evidence suggests a certain degree of protection against DNA degradation^25,26^. Such studies have been supported by Serrulla et al.^26^ who recovered 45 well-preserved brains from a Spanish civil war mass grave. In this case, the wet conditions at the level of the burials have led to poor skeletal preservation but exceptional soft tissue preservation including brains^27^. Despite brain tissues having been recovered from both forensic and archaeological environments, biomolecular research from brain tissue is extremely limited with only a handful of examples^28,29^. Therefore, the necessity of having an optimised protocol in place to allow the extraction of biomolecules from soft tissues that are resistant to degradation is highlighted.

Third, in aDNA studies, mineralised matrices such as bones and teeth are preferred, largely because early studies showed that, in comparison to skeletal remains, DNA derived from soft tissues is significantly more fragmented, has a low endogenous DNA content, and only survives under certain climatic conditions in the archaeological record^30^. As a result, most methods upstream of high throughput DNA sequencing (HTS) have been optimised for mineralised samples. However, soft tissues can be more informative than skeletal tissues, especially in palaeopathological studies, as they carry different molecular information^31^. Brain samples can facilitate omics analyses with unprecedented resolution as neurodegenerative and psychiatric disorders are affected by a multitude of factors such as pathogen-driven selection, genetics and environmental influences the signatures of which can be detected with omics analyses, specifically through lipidomics and proteomics. In addition, brain samples can play a key role in elucidating evolutionary patterns, as the brain has been shaped over the course of time^32^. Other soft tissues such as muscle and skin can offer a more direct representation of an individual’s metabolic state, including disease-related markers or evidence of exposure to toxins. Therefore, developing an extraction protocol that is optimised for soft tissues and facilitates multi-omics analyses is of paramount importance.

Therefore, we have devised an integrated two-step protocol of extracting lipids, metabolites, proteins and DNA (Fig. S1). We combined a well-established solvent-based approach that facilitates co-extraction of lipids and metabolites through phase separation by centrifugation. This results into two layers: the non-polar lipids in the top phase and the polar metabolites in the lower phase, leaving denatured protein and DNA pelleted at the bottom of the tube. The second step involves the resuspension of DNA and protein, separating the DNA from the protein-tissue pellet with a final precipitation step, leaving the DNA soluble in the supernatant. The subsequent extracts are then ready to be prepared for downstream data acquisition.

## Results

### Artificial desiccation results

Four pig brain biopsies (SS1, SS2, SS3, SS4) from the cerebral cortex were obtained and each was split into two hemispheres. Because our goal for this paper was not to observe degradation in real-time, we followed a basic desiccation protocol that has been used before in similar studies^33^. Three of the samples were desiccated at one time point, and the fourth sample was desiccated for twice the time to confirm degradation occurrence and highlight discernible differences. A total of 2 mg of cerebral cortex was collected from the frontal lobe to facilitate extraction using a) standalone (SA) protocols of DNA^12^, protein, lipid and metabolite^14^ extractions and b) our integrated (INTG) extraction protocol (Table S1). Whilst mummification by desiccation can lead to good preservation of biomolecules, previous experimental mummification studies using a range of desiccation methods have illustrated that significant DNA degradation can be measured in a range of both human and pig tissues^33–35^.

To assess degradation, we made morphological observations typically associated with artificial desiccation experiments including visible shrinkage of the cerebral cortex and decrease in percentage mass due to loss of water content (Figs. S2–S5, Table S2). On a molecular level we assessed degradation through observations within the genome and proteome, as the mechanisms underlying lipid degradation are not entirely understood, and the main product, oxidation can be a result of either a signalling process or degradation. We found approximately 15% of protein-containing modifications, of which 50% were deamidation. Upon further inspection, we identified deamidation in proteins not associated with these modifications in vivo*,* such as Syntaxin binding proteins (F1RS11) and tubulin (F1SHQ8), as well as N-acetylation at predicted residue sites in Serine- and Arginine-rich splicing factor 3 (F1RY92) within both protocols. This illustrates that modifications from both post-translational and post-mortem processes were recovered, and demonstrates that our artificial desiccation protocol successfully mimics spontaneous protein diagenesis. Despite the limitations in obtaining quantitative estimation of DNA degradation using Bayesian computation through mapDamage^36^ we employed a qualitative approach to assess DNA sequence characteristics indicative of degradation. Through visual inspection, we observed signs of deamination, fragmentation, low base quality scores and a decrease in mean sequence length, all of which indicate damaged sequences suggestive of degradation. After extraction of the desiccated tissues, DNA was quantified using a Qubit™ 4 fluorimeter (**Figure S6)**.

### DNA results

DNA recovery showed the largest deviation when compared to the SA protocol from equivalent masses of brain tissue. All four samples showed a significant increase in yield which is evident from the amount of raw and mapped reads per chromosome (Table 1) as well as the number of single nucleotide polymorphisms (SNPs) recovered (Table 2).The sequencing results for both the INTG and SA protocols can be found in Table 1. In line with increased yields of DNA, the number of raw reads following sequencing was significantly higher than the integrated protocol compared to that of the dedicated DNA extractions with an approximate average of 27 M reads compared to16M reads, respectively. However, the increased number of reads is to be expected given the higher DNA concentration recovered, but it should be noted that identical elution volumes were used to resuspend the DNA post-extraction. Furthermore, for DNA samples processed by the dedicated protocol, on average more than 60% of reads aligned to the *Sus Scrofa* genome were removed during the duplicate removal stage of data pre-processing. This is indicative of a less-than-optimal sample preparation where the PCR is dominated by a low yield of correctly formatted amplifiable library molecules.

**Table 1:** Mapping Statistics per library and mapped reads per chromosome.

	SS1 SA	SS1 INTG	SS2 SA	SS2 INTG	SS3 SA	SS3 INTG	SS4 SA	SS4 INTG
Raw reads	7,003,531	36,467,461	21,619,544	13,386,446	21,761,393	40,849,208	15,540,794	52,678,198
Mapped reads	4,496,535	22,075,298	13,667,758	9,399,508	10,680,890	26,018,098	10,290,120	38,969,751
Unique reads	**814,656**	**16,941,890**	**7,956,412**	**7,326,233**	**4,045,118**	**7,702,800**	**3,278,082**	**32,096,857**
Redundancy	81.87%	23.25%	41.78%	22.05%	62.12%	70.39%	68.13%	17.63%
μ read length	60 bp	56 bp	55 bp	68 bp%	68 bp	62 bp	69 bp	
End. DNA	11.6%	46.5%	36.8%	54.7%	18.6%	18.8%	21.0%	60.9%
Chr1	85,904	133,158	803,696	752,368	427,869	808,989	345,566	3,233,544
Chr2	49,126	1,662,529	501,769	471,091	245,168	479,456	207,966	2,155,310
Chr3	46,128	1,088,957	483,881	447,903	229,480	451,855	196,438	2,073,002
Chr4	43,105	1,059,710	414,411	384,060	212,955	408,760	175,313	1,703,578
Chr5	34,394	879,573	346,039	322,773	171,922	331,634	145,233	1,466,299
Chr6	59,542	745,787	627,085	573,367	294,955	583,271	253,859	2,713,065
Chr7	40,976	1,390,611	411,008	372,069	203,856	392,386	169,593	1,686,410
Chr8	43,164	878,092	399,120	378,586	216,363	406,545	173,414	1,604,841
Chr9	45,355	833,367	429,374	397,536	221,642	423,235	180,345	1,737,267
Chr10	23,742	899,091	235,310	213,352	119,332	225,803	97,399	949,637
Chr11	25,114	503,701	238,235	227,187	124,018	236,179	101,802	977,115
Chr12	23,523	509,816	261,811	240,418	112,750	229,708	102,836	1,197,572
Chr13	64,133	608,040	600,899	559,136	320,681	603,339	258,840	2,380,203
Chr14	47,232	1,232,094	475,958	431,745	235,702	457,671	196,264	1,963,887
Chr15	44,087	1,017,433	408,414	382,245	218,323	413,062	176,667	1,643,580
Chr16	25,458	845,872	254,141	229,567	125,801	237,558	103,267	963,285
Chr17	22,050	494,678	230,570	208,237	108,455	212,637	92,550	968,453
Chr18	19,182	506,110	191,335	176,804	95,352	184,820	78,909	801,665
ChrX	36,067	414,699	345,218	325,376	187,258	342,860	74,495	703,636
ChrY	268	724,315	2,323	2,455	1,196	2,532	10,224	89,596
MT	15,749	6,483	61,921	23,159	42,193	57,335	41,859	171,291

Each sample yielded between 7.1 and 52.7 million reads, with an average of 60% of the reads uniquely mapping to the Sus Scrofa genome. The samples consistently exhibited a substantial amount of endogenous content, ranging from 5.3% to 61.2%. The average fragment lengths varied between 55 and 69 bp. Unique mapped reads are in bold.

**Table 2:** Variant calling per sample, per chromosome.

Chromosome	RefSeq	SS1 SA	SS1 INTG	SS2 SA	SS2 INTG	SS3 SA	SS3 INTG	SS4 SA	SS4 INTG
Chr 1	NC_010443.5	0	3,506	87	274	10	73	15	22,595
Chr 2	NC_010444.4	0	4,784	126	431	5	73	14	24,844
Chr 3	NC_010445.4	2	5,298	134	314	21	117	12	28,906
Chr 4	NC_010446.5	4	3,104	66	239	27	69	22	16,470
Chr 5	NC_010447.5	6	3,230	224	400	43	152	100	19,317
Chr 6	NC_010448.4	1	5,391	149	441	17	106	16	19,648
Chr 7	NC_010449.5	2	2,372	75	179	9	51	11	19,899
Chr 8	NC_010450.4	2	1,793	150	268	24	98	45	14,880
Chr 9	NC_010451.4	1	2,392	172	353	30	107	25	17,676
Chr10	NC_010452.4	4	2,060	118	203	21	142	23	12,010
Chr 11	NC_010453.5	0	1,638	27	125	2	50	0	10,906
Chr 12	NC_010454.4	0	4,977	112	360	18	60	11	23,295
Chr 13	NC_010455.5	1	1,879	93	171	12	54	18	13,213
Chr 14	NC_010456.5	0	2,915	77	247	6	46	8	22,075
Chr 15	NC_010457.5	8	2,292	100	209	17	55	20	12,205
Chr16	NC_010458.4	12	1,280	121	202	78	125	62	7,703
Chr 17	NC_010459.5	1	2,501	78	186	6	40	19	13,331
Chr18	NC_010460.4	0	1,564	37	117	1	29	3	9,915
Chr X	NC_010461.5	3	814	29	75	0	18	0	1,440
ChrY	NC_010462.3	0	30	2	3	0	1	509	2,009
MtDNA	NC_000845.1	173	182	177	174	19	20	20	1,440

SA: Standalone Protocol, INTG: Integrated Protocol.

The most notable difference between the two protocols was observed during variant calling. The integrated protocol yielded a much higher number of SNPs across all chromosomes. Manual inspection of regions that were mutually covered by aligned sequencing reads exhibited a greater depth of coverage favouring the integrated protocol (Table 2). We further analysed samples SS2 (SA)/SS2 (INTG) which have largely comparable numbers of aligned reads (in total and per chromosome) and found a significantly higher number of SNPs obtained from the integrated protocol. We compared the read-to-SNP ratio of the unfiltered VCF files and the percentage of SNPs passing filtering (MQ < 30, DP > 5, QUAL30, QD < 2.0, SQR > 3.0, FS > 60.0, MQRankSum < − 12.5, ReadPosRankSum < − 8.0) was much higher from the INTG protocol. For example, sample SS2 (SA) has a higher number of aligned reads overall and following read duplicate removal compared to its equivalent SS2 (INTG) (Table 1). Despite the higher number of aligned reads the number of SNPs passing quality filtering was more than double for the INTG protocol. Furthermore, the percentage of reads passing quality filtering was much higher (X% vs Y%) for the INTG approach. Due to the differences in the number of raw reads per library, the remaining samples could not be directly compared. However, we normalised the data and analysed SNP-to-aligned read ratio, again finding that it was significantly higher in favour of the integrated protocol. Our results suggest that, when considered holistically, DNA preparation using the integrated protocol was more successful compared to the standalone approach throughout all stages, from sample preparation to computational processing.

### Protein results

**Figure 1:**
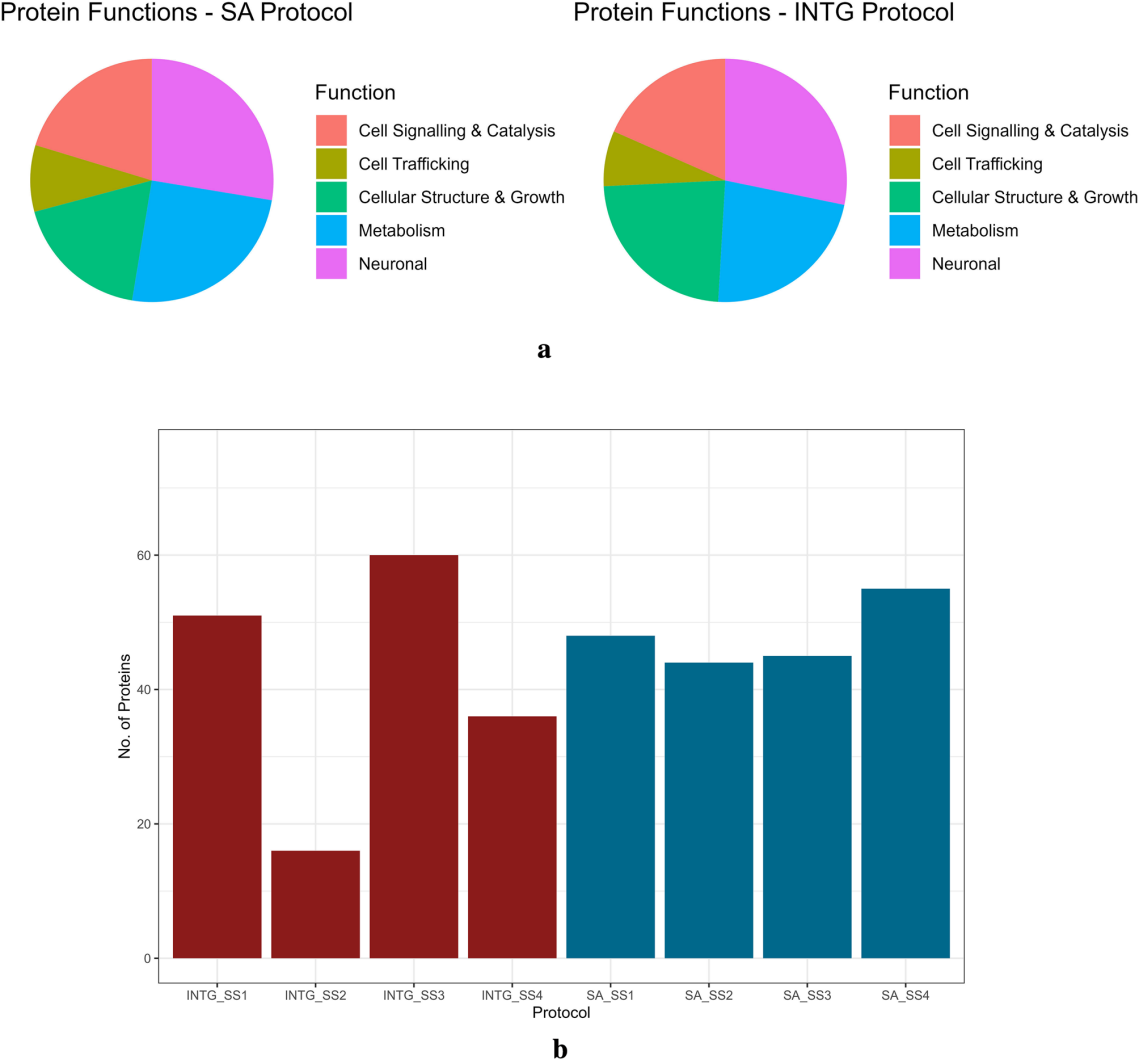
(**a**): Allocated categories of peptide sequence matches for each protocol. Labelled as Neuronal are the matches where the protein or isoform is specifically located within the brain or central nervous system. (**b**): Protein Recovery between INTG and SA Protocols. 163 proteins and 192 proteins were identified in all INTG (blue) and SA (red) samples collectively.

**Figure 2:**
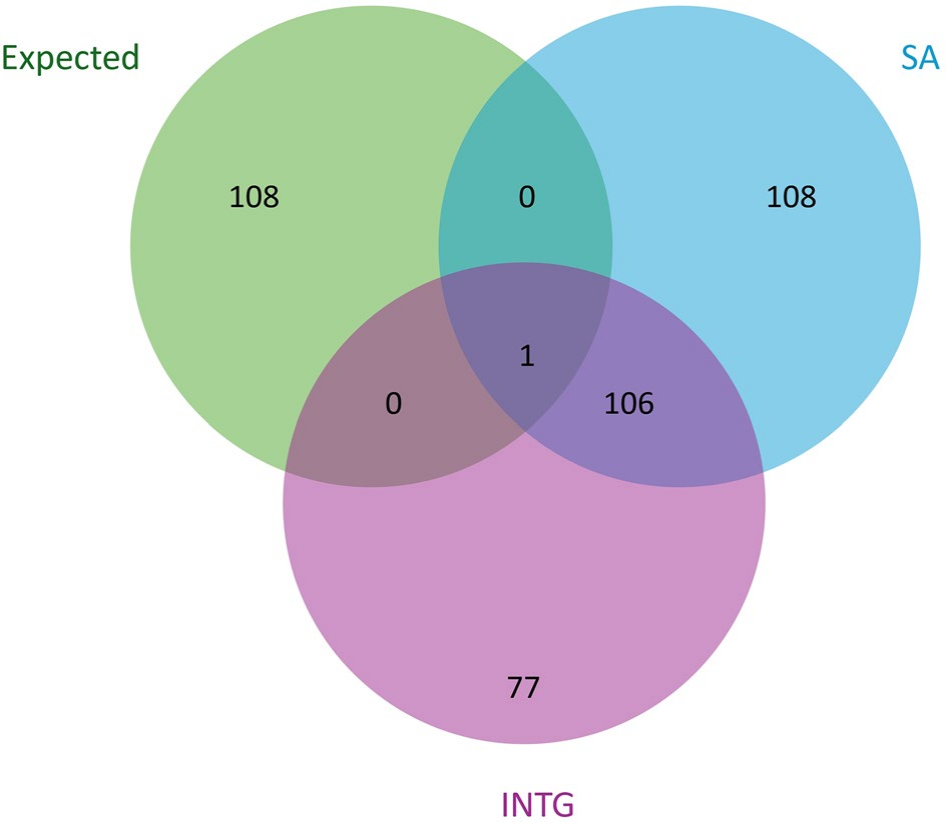
Comparison of enriched protein expression between protocols. Expected (green) represents protein where expression is regionally enhanced within the cerebral cortex of *Sus Scrofa* (n = 109). The list of expected proteins was acquired from the Human Protein Atlas^39^.Only proteins identified with high confidence across all samples were retained for comparison with a total of 215 and 184 proteins for SA and INTG respectively with a single expected protein recovered by both methods.

The results are summarised in Fig. 1a,b. Following shotgun proteomics, we validated peptide sequence matches (PSMs) of raw files through MaxQuant^37^ search using MSStats^38^ for processing and summarisation, before comparing SA with INTG protocols for both normalised peak intensities of total proteins discovered (Fig. S7) and categorising their general functions (Fig. 1a). Both protocols show that most proteins are related to neuronal and metabolic functions, which is expected within brain samples. Furthermore, proteins and isoforms specific to the brain and nervous system were the dominant class in each protocol, showing a high degree of uniformity. While there were more PSMs in the SA protocol than in the INTG post-processing, we found that INTG SS2 had significantly fewer proteins (Fig. 1b) recovered and at lower peak intensities (Figs. S7, S8) than the other three INTG samples, indicating a loss or disruption of the sample in the process. In contrast, a greater overall recovery of individual protein abundance in SS1-SS3 samples (Fig. S8), such as with the ubiquitous tubulin, spectrin, alpha (axon actin-ring cytoskeleton) and syntaxin binding protein 1 (neurotransmitter release in synaptic vesicles), further validates the comparability between protocols. To further asses the recovery of peptides derived from regionally enriched protein expression^39^ in the cerebral cortex of *Sus Scrofa* (n = 109), we first filtered the identified proteins to remove contaminants, with any protein that did not have at least one unique peptide removed from the dataset. Data was filtered to retain proteins identified with high confidence according to an FDR below the 0.01 threshold. Only proteins identified with high confidence (FDR threshold = 0.01) were retained (Fig. 2). Venn diagrams were generated using jvenn^40^.

### Lipid and metabolite results

**Figure 3:**
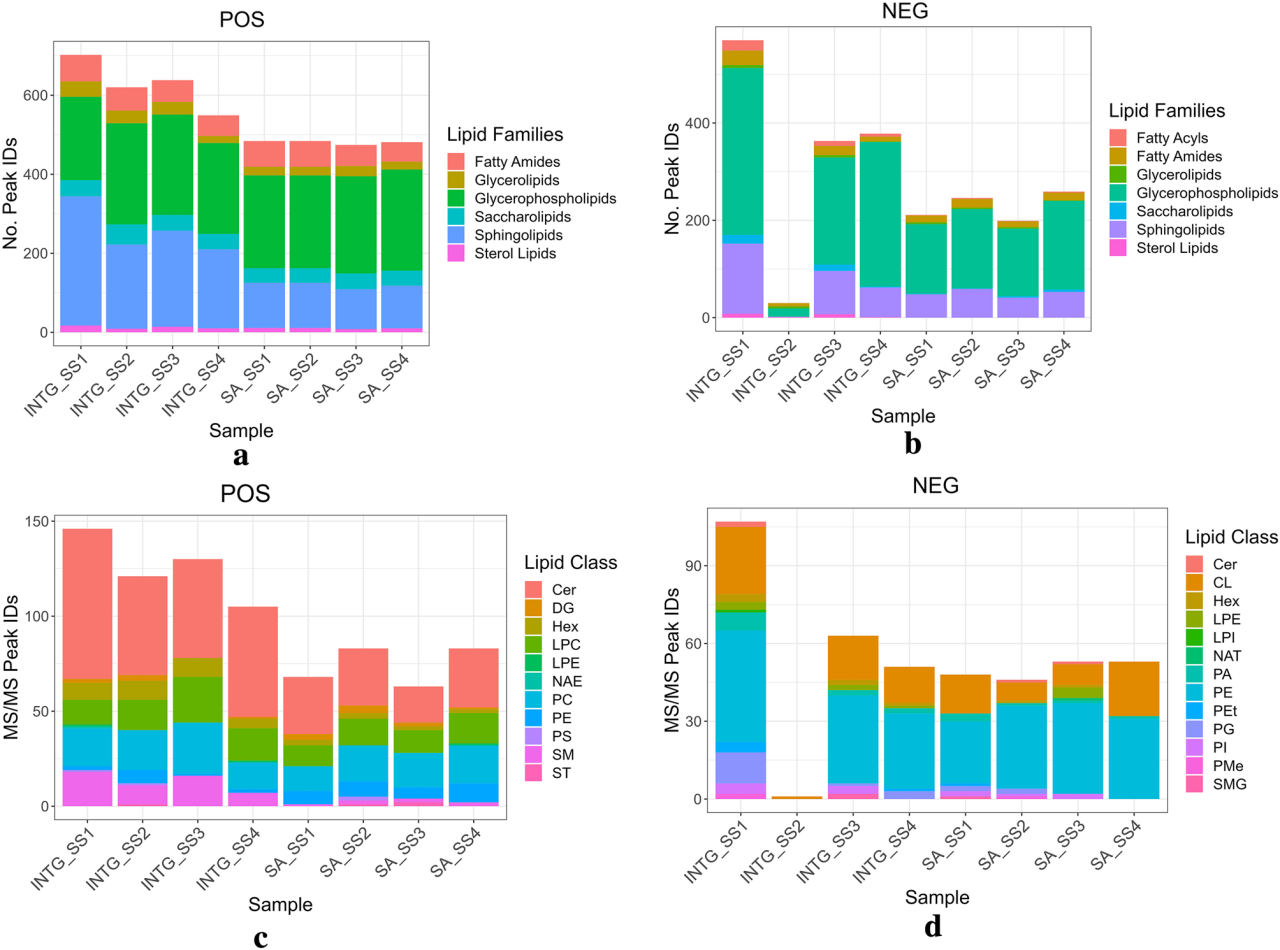
(**a**, **b**)**:** Lipid families found in both MS and MS/MS. (**c**, **d**): Individual lipid classes of MS/MS matches.

**Figure 4:**
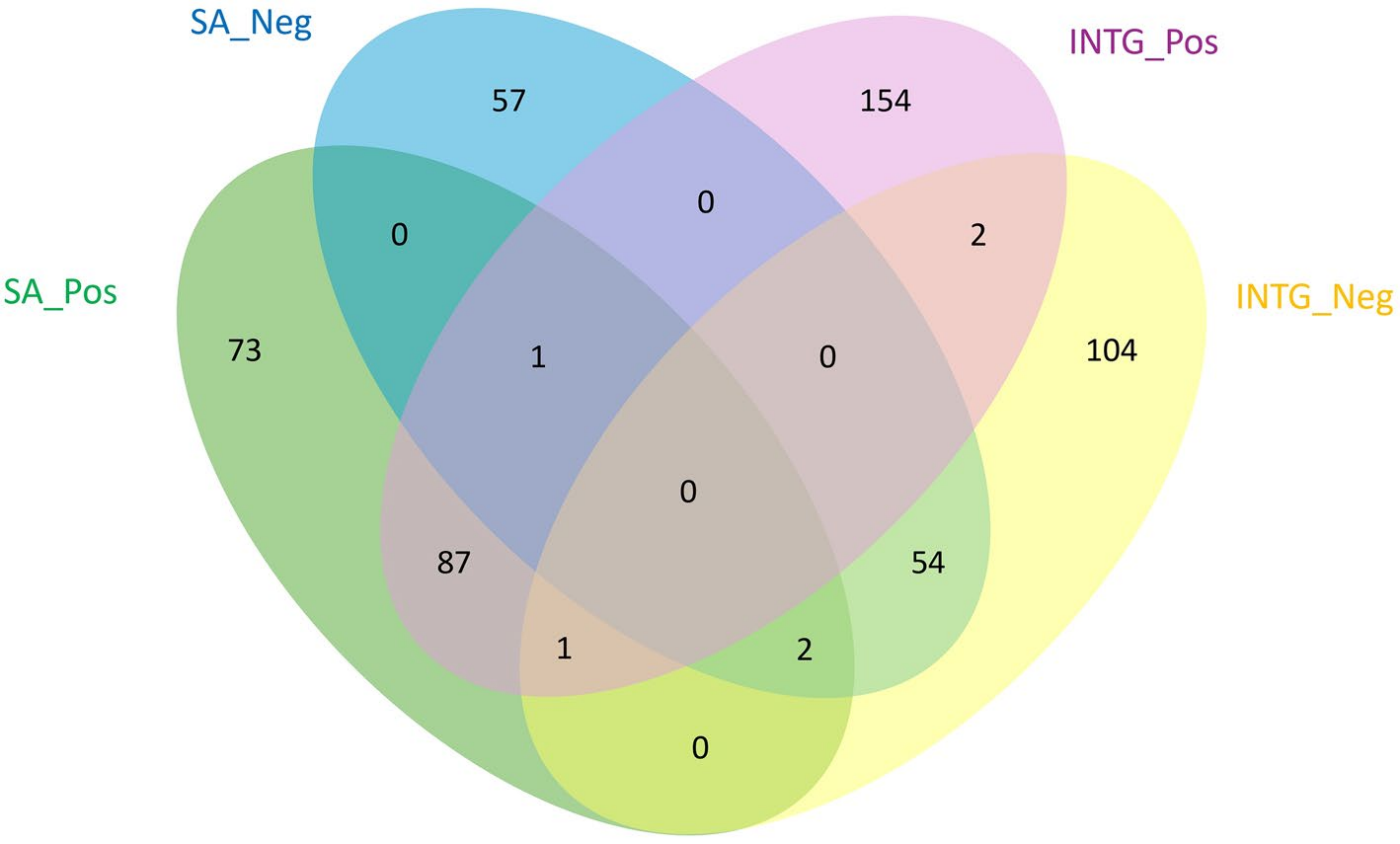
Comparison of MS2 acquired lipid identifications. In total, SA and INTG generated 276 and 445 MS2 acquired identifications in positive mode and 196 and 221 in negative mode respectively. INTG showed approximately double the amount of lipids that were acquired exclusively in both positive and negative modes when compared to the SA protocol.

The initial lipid spectral identifications showed that, in general, the INTG protocol recovered more peaks across both mass spectrometric modes than Bligh and Dyer^14^, with a significant increase in a positive mode (Fig. S9). When examining the lipid family composition (Figs. 3a, b), the protocols are highly comparable and consistent with current brain lipid literature. The predominance of glycerophospholipids in both modes is expected due to their being the main constituents of neuronal cell membranes^41^ while the significant abundance of sphingolipids is commonly found within the brain due to their roles in neurogenesis and synaptogenesis^42^. These general observations of tissue specificity and lipid recovery are similarly reflected when discriminating into lipid classes (Figs. 3c, d), where sphingomyelin and its biosynthetic ceramide precursors, both highly enriched in oligodendrocytes and myelin, consist of the majority of positive mode identifications. In contrast, the membrane-based phosphatidylethanolamine (PE) and its derivatives dominate the negative mode, while also elucidating cardiolipin, a hallmark of inner mitochondrial membrane that is predicted to be abundant within the brain^43^. When examining the lipid subclasses composition (Fig. 4), we observed approximately double the amount of lipids with the INTG protocol in positive mode compared to the SA protocol, where these lipids predominantly account for ceramides, monoacylglycerophosphocholines and sphingomyelins. In negative ionisation mode, diacylglycerophosphates and monoacylglycerophosphoglycerols account for most of the exclusive identifications with INTG. Conversely, phosphatidylethanolamines mainly account for compounds identified exclusively with SA in both ionisation modes.

The only sample which had significantly less recovery when using the INTG protocol was SS2 negative mode lipids (Figs. 3b, d), which further supports loss of sample during phase separation or handling. However, statistically, there was virtually no difference between protocols in terms of peak identifications (Fig. S9).

**Figure 5:**
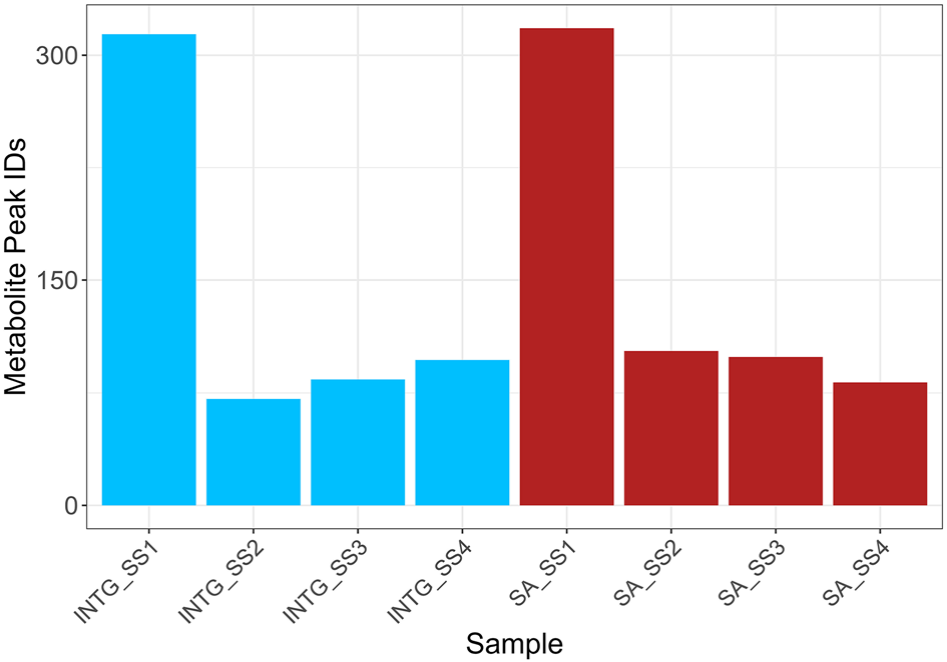
Brain Lipids and Metabolites obtained from INTG (blue) and SA (red) protocols.

**Figure 6:**
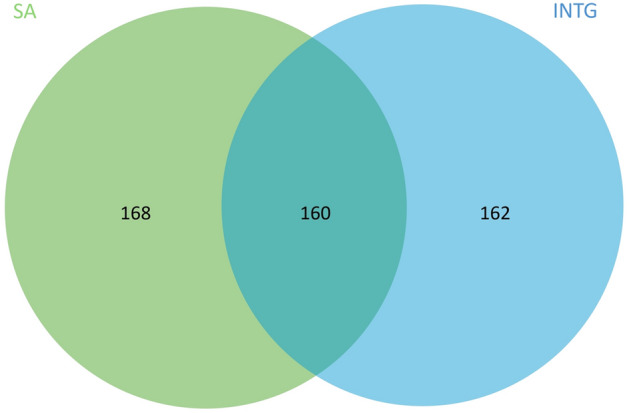
Comparison of MS1 acquired metabolite identifications. SA and INTG protocols generated 328 and 322 identifications respectively with approximately 50% of the matches being concordant between methods. Due to lack of acquired MS2 data for both protocols, we compared the total number of compounds with suggested identifications.

Statistical analysis of small molecules and metabolites showed no significant difference between the Integrated and traditional protocols used, with comparable recovered MS peak identifications (Figs. 5, S9). Due to the lack of acquired MS2 data for both protocols, we compared the total number of compounds with suggested identifications (Fig. 6). Therefore, it was not possible to focus our comparison on reference-matched identifications as was conducted with the lipids. Identifications were excluded if they did not display 100% coverage across all samples within each protocol (n = 4) to minimise any bias from differing metabolism between animals. We observed a comparable recovery of molecular classes in both protocols with the most abundant class of molecules shared by both protocols belonging to amino acids. However, we are unable to distinguish if this is a result of natural protein turnover in vivo or a result of protein degradation. Despite this, it is logical to assume that this is a combination of protein metabolism and degradation resulting from the treatment of the tissues prior to extraction. Differences between protocols are generally attributed to variation between a small number of amino acids and saturated fatty acids. The exact reasoning is unclear as both protocols rely on MeOH for the recovery of polar metabolites. However, the ratio of MeOH to non-polar solvent does differ, and hence the final concentration of MeOH differs between protocols and may explain the slight difference in the recovery of polar metabolites.

## Discussion

This proof-of-concept paper demonstrates for the first time the successful implementation of a novel multi-omic integrated protocol for the simultaneous extraction of DNA, proteins, metabolites and lipids from a single degraded sample. This approach increases the acquisition of omics data from a single sample while reducing time, associated costs, and more importantly, sampling requirements (which is of paramount importance, when finite or archival and clinical FFPE samples are processed).

Four porcine brain samples were artificially degraded for up to three months at 37 °C and subsequently sub-samples were processed using well-established standalone protocols for the individual extraction of DNA^12^, proteins, lipids and metabolites^14^. Equivalent sub-samples of the same four samples were also processed using our integrated protocol. Comparisons between the integrated and standalone protocols demonstrate that our multi-omic protocol is either comparable or superior to the single-omic protocols. Both protocols generated a substantial number of raw reads, with the SA protocol producing between 6 and 21 million reads, while the INTG protocol produced between 13 and 52 million reads. Discrepancies in the raw data could be attributed to differences in DNA concentration, pooling and sequencing bias. However, when we normalised the results to account for biases, we observed consistently better results in favour of the integrated protocol for all samples (except for SS1, which exhibited more than twice the proportion of mapped reads compared to raw reads (Table 1), but this did not translate into higher rates of SNP calling). Additionally, when mapping to the *Sus Scrofa* genome, the samples processed with the INTG protocol showed a significant percentage of high-quality SNP calls per sample compared to the dedicated protocol (Table 1), even when the number of aligned reads was lower than the equivalent SA sample (Tables 1 and 2). Aligned reads per chromosome generally showed comparable or better results between the two protocols, except for a few instances where the SA protocol exhibited more Y-chromosomal reads (Table 1). In addition, there was increased depth of coverage which is of great significance for applications in ancient and forensic fields, as it decreases the need for additional target enrichment. Similarly to the DNA results, protein recovery was also comparable or higher in all four samples apart from sample SS2 which is likely the result of a loss of sample during phase separation or handling. Organic molecule, metabolite and lipid recovery was consistently comparable among all samples, with the INTG protocol recovering more identifications in both positive and negative modes during lipidomic analysis.

So far, biomolecular analyses of degraded brain samples have been extremely rare, with only two successful examples documented in the literature^27,28^ following singe-omic approaches. Additionally, one study produced dubious results^29^ due to the lack of adherence to strict anti-contamination criteria. With rare exceptions, the standardised and comprehensive analysis of ancient, forensic, mummified and other heavily degraded brain tissues has been notably lacking on a large scale. This gap signifies a vast reservoir of unexploited tissue resources that could significantly contribute to the exploration of brain evolution. As a consequence, contemporary research on human brain evolution^44–47^ has predominantly relied on modern samples, primarily focusing on the genomic level. Computational approaches, cross-species comparative genomics, as well as population genetic-level approaches, have been instrumental in exploring the diversity of the human genome. Notably, these studies have highlighted the role of Transposable Elements (TE) as influential drivers of human brain evolution^32^.

However, pathogenesis encompasses a complex interplay of various regulatory elements such as proteins, metabolites, lipids, genes and epigenetic features such as DNA methylation and histone modifications. Considering the brain is abundantly found in mummified remains and is resistant to degradation due to its high lipid content, multi-omics studies including both ancient and modern data are critical in providing insights into the intricate dynamics underlying brain evolution such as genetic, epigenetic and biochemical factors. Furthermore, it is possible to unravel the mechanisms behind well-documented brain preservation in the archaeological record.

By successfully characterising biomolecules from degraded brain samples, we systematically address the limitations posed by degraded soft tissues, thereby challenging preconceived notions regarding the applicability of such samples in biomolecular and biomedical research and re-assessing their value within the context of brain evolution and pathogenesis. In particular, the protocol’s ability to reduce processing time and material requirements while also minimising biases associated with sample preservation variations, which are responsible for deviations in results and a lack of authentication and replication, enhances its practical utility. Processing a single sample under the same protocol and storage conditions, has the potential to help with noise reduction and artefact reduction for downstream bioinformatic applications by facilitating top-down approaches to simultaneous integration and dimensionality reduction of both NGS and MS datasets, which will further improve robustness for subsequent machine learning methods. This quality is especially valuable in studies involving finite or archival samples, where obtaining meaningful data can be challenging.

Additionally, we underscore the brain’s significance as a model tissue and lay the groundwork for future investigations into various tissues. The initial focus on brain tissues serves as a strategic starting point acknowledging the inherent challenges associated with these samples. As the protocol proves its efficacy in the brain, the potential application to other tissues becomes evident, promising a versatile tool for multi-omic analyses in diverse biological contexts.

## Methods

*Integrated Protocol Overview:* The integrated protocol was validated using four artificially desiccated samples (SS1, SS2, SS3, SS4) against dedicated well-established and widely used extraction protocols for DNA, protein, lipids and metabolites. Subsequent extracts were processed for individual downstream workflows, and data acquisition performed via high throughput sequencing (HTS) for DNA and liquid chromatography with tandem mass spectrometry (LC–MS/MS) for protein, lipids and metabolites. For both the INTG and SA protocol for DNA, vortexing and ultrasonication were avoided to prevent fragmentation of the DNA. This is important in this study as we want to ensure this protocol is suitable for forensic and archaeological studies and case work. A schematic representation of the workflow is presented in Fig. S1.

### Step 1: Organic molecule separation

For our INTG protocol, we opted for a relatively large volume of extract as we did with the standalone protocol^14^. The initial ratio of methyl-tetr-butyl ether (MTBE) and methanol (MeOH) (10:3 v/v) based lysis remains true to the original description^16^. However, we included 0.01% butylated hydroxytoluene (BHT) in our MTBE:MeOH mixture, to minimise oxidation of lipids. Furthermore, we incorporated the use of 0.1% ammonium acetate (AA) to induce phase separation^17^ while aiding precipitation of insoluble material including DNA and protein. Lipids and metabolites were first extracted using 4 mL MTBE:MeOH in a 10:3^17^ ratio with a 60 min incubation step at 4 °C with gentle agitation in a shaking incubator. Phase separation was induced with 770 μL 0.1% AA (10:3:2.5 (v/v/v)) to aid precipitation of insoluble material, including protein and DNA. The upper layer, containing the non-polar lipids, and the lower layer, containing the polar metabolites, were then decanted into fresh glass tubes for drying and resuspension. Extracts were then dried under a gentle flow of nitrogen at 40 °C and re-suspended in either 200 μL MTBE containing 0.01% BHT (lipids) or 200 μL of 80% aqueous acetonitrile (ACN) with 0.1% formic acid (FA) (metabolites) and submitted to the Bio-MS core facility at the University of Manchester for data acquisition.

### Step 2: DNA and Protein separation

The resultant pellets from the original extraction tubes were left to air dry for 5–10 min. The pellets were then resuspended in 500 µl of 5% sodium dodecyl sulfate (SDS) based lysis buffer (pH8) and left to incubate at room temperature for 30 min with gentle agitation. Following centrifugation at 1500×*g* for 10 min to pellet any insoluble material, the supernatant was transferred to 2 mL DNA Lobind tubes (Eppendorf) and 250 μL ammonium acetate (AA) (7.5 M) was added for a final concentration of 2.5 M. Following the addition of AA, samples were left to incubate at room temperature for 15 min and the protein was pelleted by centrifugation at 16,000×*g* for 10 min. The resultant supernatant, containing DNA, was then suspended in five volumes of spiked Qiagen PB buffer^12^ and purified by MinElute (Qiagen) with the addition of reservoirs for larger sample volumes^12^, followed by two rounds of elution with 30 µL elution buffer (Qiagen EB). Extracts were stored at − 20 °C until library preparation and sequencing at the Genomics Facility of the University of Manchester for data acquisition.

The remaining protein pellet was resuspended in 500 µL of a protein lysis buffer consisting of 5% SDS, 50 mM Tetraethylammonium bromide (TEAB) and LC–MS grade H_2_O, (pH 7.5). All samples were reduced with dithiothreitol (DTT) at a final concentration of 5 mM, for 10 min at 60 °C and allowed to cool to room temperature. Alkylation was facilitated with iodoacetamide (IAM) at a final concentration of 15 mM with a 30 min incubation at room temperature in the dark, followed by quenching with DTT. Samples were then centrifuged at 14,000×*g* for 10 min to pellet insoluble debris and the resultant clarified lysate was transferred to a fresh protein LoBind tube (Eppendorf).

Lysates were quantified on a Qubit™ 4 fluorimeter, with the Protein Broad Range assay kit (Invitrogen). Trypsin digestion was performed using the S-Trap column digestion protocol^48^ (ProtiFi). All subsequent binding, washing and elution steps were followed by centrifugation at 4000×*g* for 2 min. Samples were diluted to 1 mg/mL and 50 µL (containing 50 µg) was acidified with 5 µL of 12% aqueous phosphoric acid. The total 55 µL of acidified sample was loaded onto the columns with 330 µL S-Trap binding buffer (90% MeOH, 100 mM TEAB, PH 7.1). One wash with MTBE, followed by four washes with S-Trap binding buffer, was performed. Lyophilised trypsin was dissolved in 50 mM TEAB and loaded onto the columns (final protease: protein ratio of 1:10) and incubated for 60 min at 47 °C. Three elution steps were performed with 65 µL of 50 mM TEAB, 65 µL of 0.1% aqueous Formic Acid (FA) and 30 µL of 30% aqueous acetonitrile (ACN) containing 0.1% formic acid (FA). Samples were further desalted using Oligo™ R3 reversed-phase resin (Thermo Scientific) and eluted twice in 50 µL of 30% aqueous ACN containing 0.1% FA. Samples were dried by SpeedVac and submitted to the Bio-MS core facility at the University of Manchester for data acquisition.

### Supplementary Information

Supplementary Information 1.

Supplementary Information 2.

Supplementary Information 3.

Supplementary Information 4.

## Data Availability

Genomic data are curated at the European Nucleotide Archive under project accession number PRJEB61253. Proteomics data are available via ProteomeXchange with identifier PXD047606. Protein, Lipid and Metabolite Datasets are provided as Additional Files.
