# Supplementary material for: A novel integrated extraction protocol for multi-omic studies in heavily degraded samples
Source: Sci Rep. 2024 Jul 30;14:17477. doi: 10.1038/s41598-024-67104-8 (PMC11289452; doi:10.1038/s41598-024-67104-8)
Supplement: Supplementary file 4 — Supplementary Information 4. [file 41598_2024_67104_MOESM4_ESM.pdf]

A novel integrated extraction protocol for multi-omic studies in heavily degraded samples

Byron Boggi[1], Jack, D.A Sharpen[1], George Taylor[2] and Konstantina Drosou*[1,3]

[1] Division of Cell Matrix Biology and Regenerative Medicine, University of Manchester, Manchester, M13 9PL, UK

[2] Faculty of Biology, Medicine and Health, Research and Innovation, Manchester, University of Manchester, Manchester M13 9PG

[3] Manchester Institute of Biotechnology, University of Manchester, Manchester, M1 7DN, UK

Byron Boggi: byron.boggi@manchester.ac.uk
Jack Sharpen: jack.sharpen@manchester.ac.uk
Geroge Taylor: george.taylor-2@manchester.ac.uk

*Corresponding author: konstantina.drosou@manchester.ac.uk

Supplementary Information

1. Overview of Integrated Extraction Protocol (INTG)

The integrated extraction protocol is a two-step process that facilitates the co-extraction of lipids and metabolites through phase separation, followed by extraction of protein and DNA from the pellet. A schematic workflow of the process is found in **Fig. S1**.

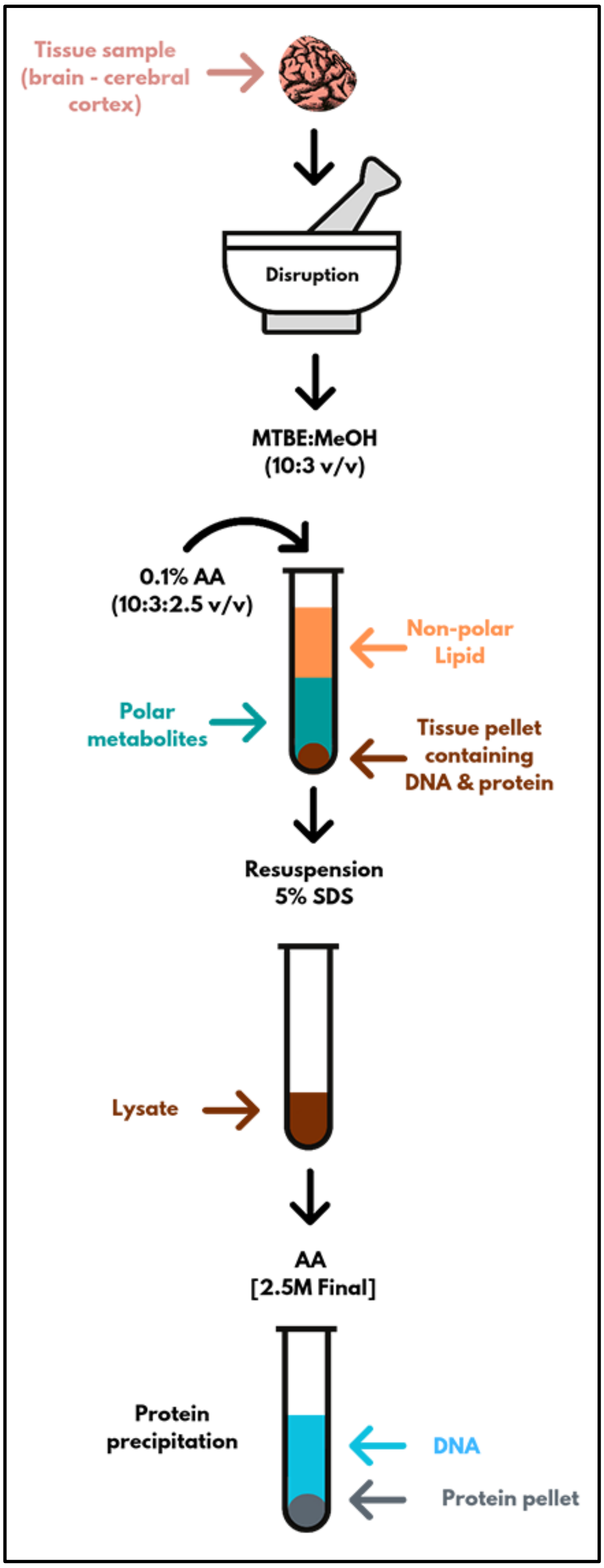

Figure S1: Schematic Representation of Integrated Protocol (INTG) Workflow.

2. Sample Selection

We used four whole porcine brain hemispheres (SS1, SS2, SS3, SS4) as analogues for human tissue to simulate a degraded environment (**Table S1**). Pigs have been routinely used as analogues in human metabolic disease research due to similarities in anatomy, development, function and neurophysiological processes[1]. Additionally, the use of pig tissue as a human analogue is well-established within forensic studies[2,3] to measure genomic[4], proteomic[5] and metabolomic[6] degradation. As such they are the ideal samples for our artificial degradation experiments. Porcine brains were acquired from a local, organic butcher. Carcasses were delivered to the butcher shortly after slaughter and excerebrated. As the meat was for human consumption and the brains are usually discarded by the butcher due to lack of customer interest, no ethical approval was required for the use of these tissues in the present study (https://www.gov.uk/guidance/meat-products-sell-them-legally-in-england).

3. Sample preparation

Brains from agriculturally-reared pigs (*Sus scrofus domesticus*) aged between 4-6 months were chosen as human analogues. Brains were separated into individual hemispheres following collection from a local butcher, with the donor animals having been deceased for less than 3 hours prior to collection. A total amount of 30mg of cerebral cortex biopsies were collected from the frontal lobes (**Table S1**). Samples were then suspended in 500µL of 50mM Tris-HCl, (PH8) and briefly vortexed to remove any sand particles stuck to the tissue. Samples were then incubated at -80˚C for 60 minutes and ground to a fine powder using a mortar and pestle on dry ice to prevent the sample defrosting and sticking to the mortar. For each protocol described in this study, 30mg of powdered sample was aliquoted and stored at -80˚C until required.

4. Artificial degradation

Artificial desiccation took place in incubators set at 35-40°C and 10-15% humidity, both of which were monitored with a digital thermometer/hygrometer to maintain ambient conditions (**Table S2**). Four brain samples (SS1-SS4) were placed on birchwood canvas panels and left either exposed or fully covered in desert sand to desiccate over the following timepoints: Samples SS1-SS3 were left for 28 days, and sample SS4 was left for 60 days with the rate and scale of desiccation measured by percentage mass loss of each brain (**Figures S2-S5**, **Table S2**) After desiccation, dried brains were collected within 1.5mL microtubes (Eppendorf) and stored at -80°C for subsequent extraction.

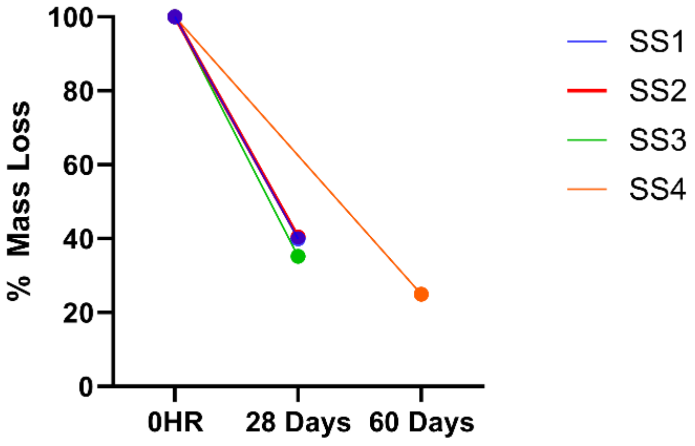

Figure S2: Loss of pig tissue mass during desiccation timepoints.

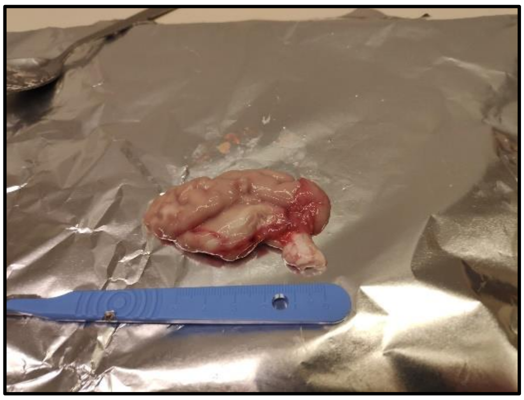

Figure S3: Brain desiccation at 0HR

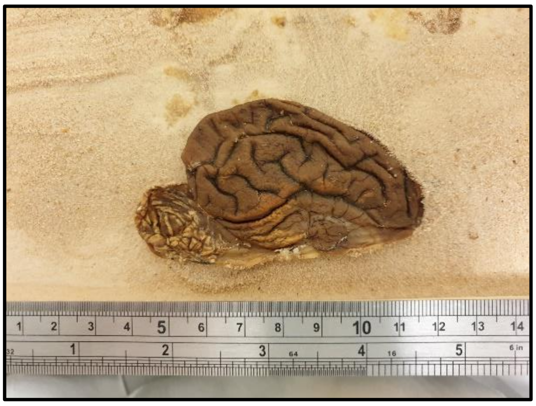

Figure S4: Brain desiccation at 28 days

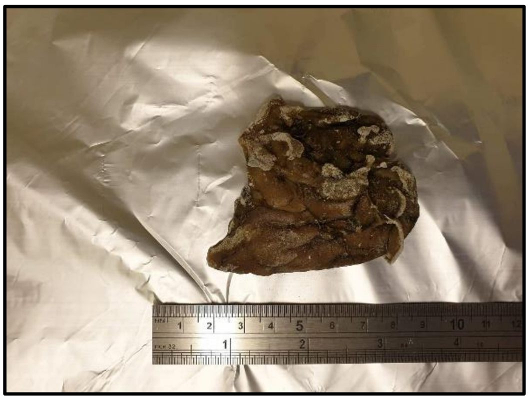

Figure S5: Brain desiccation at 60 days

5. Anti contamination regime

Our anti-contamination regime followed stringent criteria frequently employed in the ancient DNA (aDNA) field[7]. Artificial desiccation procedures were conducted in a dedicated mummification lab with restricted access, whereas post-processing of samples took place in a separate modern DNA lab. All surfaces, including dissection benches, were cleaned with 5% bleach and 70% ethanol, and all utensils and equipment were treated with DNA-Away (Molecular Bioproducts) before and after use. Items such as test tubes were UV irradiated (254nm, 120,000 μJ cm^{-2} for 2 × 5 minutes, with 180° rotation between the two exposures on a Stratalinker UV cross linker) before use. Aqueous solutions excluding organic solvents due to their volatility were similarly irradiated for 15 minutes.

Brain biopsies were collected, using personal protective equipment including forensic coveralls, face masks, hair nets, goggles, boot covers and two pairs of sterile gloves. Prior to all extractions, glassware such as Pasteur pipettes, tips and tubes, as well as glass tubes with their corresponding polytetrafluoroethylene-lined caps were decontaminated in Chemgene and ultrapure water followed by UV irradiation as described above.

DNA extractions were carried out in a Class II biological safety cabinet and sequencing libraries were prepared in a laminar flow cabinet. All extractions were accompanied by two extraction blanks (normal extraction but without sample material). All organic solvent-based extractions were performed in a fume hood while wearing appropriate protective equipment, including chemically resistant nitrile gloves, protective goggles, hair nets and disposable face-masks to limit risk of skin contact.

6. Standalone Extractions

6.1 DNA extraction

The dedicated extraction protocol used for comparison in this study is a well-established and widely adopted extraction protocol optimised for aDNA that makes use of commercially available silica spin columns[8]. As this protocol was originally designed for mineralised tissues and relies on a simple 0.5M EDTA and Proteinase K-based digestion buffer, we modified our lysis buffer composition with the addition of 1% SDS in 50mM Tris-HCl (pH8) to account for the high lipid content in brain tissue. Furthermore, 5M sodium acetate (NaOAc) and 5M sodium chloride (NaCl) were added to Qiagen's binding buffer (PB) to improve binding of ultra-short fragments of DNA to the silica membrane as described previously[9]. Samples were lysed overnight at room temperature. No fragmentation step was performed during extraction and samples were lysed in 500μL of lysis buffer containing 20μL proteinase K (20 mg/mL). An overnight incubation (18 hours) was performed at room temperature with gentle

agitation in a shaking incubator prior to purification. Following extractions, DNA concentration was quantified by Qubit™ 4 fluorimeter (**Figure S6**).

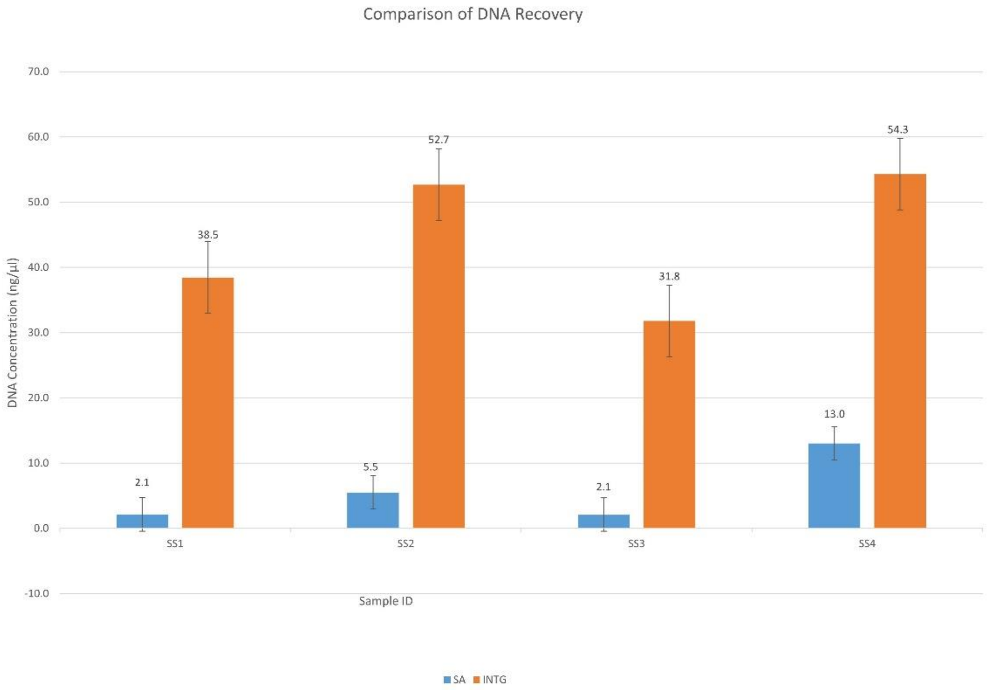

Figure S6: Qubit values after DNA extractions.

6.2 Protein Extraction

A total of 30 mg of cerebral cortex were suspended in 500µL of lysis buffer consisting of 5% SDS, 50mM TEAB and LC-MS grade H_2O, pH7.5. Lysis was facilitated using the Covaris LE220+ ultrasonication system. Subsequent reduction, alkylation, digestion and desalting followed the exact same procedure detailed in the 'Integrated protocol overview' section in the main manuscript.

6.3 Lipid and Metabolite Extraction

For the dedicated lipid and metabolite extraction protocol, a chloroform ($CHCl_3$) and methanol (MeOH) based mixture (2:1) was selected due to its long-standing popularity for lipid and metabolite extraction[10]. Here, phase separation is induced with H_2O, with non-polar lipids collected from the lower $CHCl_3$ phase and polar metabolites collected from the upper MeOH phase.

Samples were suspended in 4mL of ice-cold chloroform:methanol (2:1) containing 0.01% butylated hydroxytoluene (BHT) and vortexed in 3 x 10-second rounds. Phase separation was induced with the addition of 1mL H_2O followed by another 3 x 10-second rounds of vortexing and centrifugation at 1,500xg for 30 minutes. Both the upper methanolic phase and the lower organic phases were transferred to fresh round-bottom glass tubes in preparation for drying. The remaining tissue pellets were resuspended in fresh CHCl3:MeOH and the process was repeated twice more, with each phase being decanted and pooled. Sodium sulphate was then added to the lipid extracts until a visible snowstorm effect was seen to remove any H_2O contamination. Extracts were then dried and resuspended as described in the integrated (multi-omic) extraction section.

7. Data Acquisition

7.1 DNA Library Preparation and Shotgun Sequencing

Eight dual-indexed NGS libraries were prepared according to previously established protocols modified for degraded samples by omitting the A-tailing step and size selection[11,12] ,including two library and one extraction negative controls. The negative controls were included along the entire workflow to monitor potential background contamination. A total of 24 cycles of indexing PCR was performed using the IS5 (5' - AATGATACGGCGACCACCGA – 3') and IS6 (5' - CAAGCAGAAGACGGCATACGA – 3') primers. Indexed libraries were quantified by quantitative PCR (qPCR), on a Roche LightCycler 480 using Sybr Green I Master kit. Quantification was further performed using fluorimetry on a Qubit 4.0 using the Qubit dsDNA HS Assay Kit. The length distribution was assessed using Agilent's Bioanalyzer 2100 using the High Sensitivity DNA kit. Libraries were purified using the MinElute Purification kit (Qiagen). Negative controls were not sequenced as their concentration and size distribution was indicative of adapter dimers, and no other identifiable amplicons were detected. Libraries were combined in an equimolar pool (10nM final concentration) of eight libraries and submitted to The Genomic Technologies Core Facility (University of Manchester) for sequencing on an Illumina HiSeq 4000. Sequence data are curated at the European Nucleotide Archive under project accession number PRJEB61253.

7.2 Protein Analysis, Nano-LC-MS/MS

A 2µL protein sample aliquot was loaded into the end of a 5µL loop and reversed flushed on to the analytical column (Waters nanoEase M/Z Peptide CSH C18 Column, 130Å, 1.7µm, 75µm x 250mm). The separation was performed on a Thermo RSLC system consisting of an NCP3200RS nano pump, WPS3000TPS autosampler and TCC3000RS column oven, configured with buffer A as 0.1% formic acid in water and buffer B as 0.1% formic acid in acetonitrile. The analytical column was connected to a

Thermo Exploris 480 mass spectrometry system via a Thermo nanospray Flex Ion source. The capillary was connected to a fused silica spray tip with an outer diameter of 360µm, an inner diameter of 20µm, a tip orifice of 10µm and a length of 63.5mm (New Objective Silica Tip FS360-20-10-N-20-6.35CT) via a butt-to-butt connection in a steel union using a custom-made gold frit (Agar Scientific AGG2440A) to provide the electrical connection. The nanospray voltage was set at 1900V and the ion transfer tube temperature set to 275°C. Spectral data was acquired in an independent manner and using a fixed cycle time of 2 seconds, an expected peak width of 15 seconds and a default charge state of 2. Full MS data was acquired in positive mode over a scan range of 300 to 1750 Th, with a resolution of 120,000, a normalised AGC target of 300% and a max fill time of 25mS for a single microscan. Fragmentation data was obtained from signals with a charge state of +2 or +3 and an intensity over 5,000 and were dynamically excluded from further analysis for a period of 15 seconds after a single acquisition within a 10ppm window. Fragmentation spectra were acquired with a resolution of 15,000, a normalised collision energy of 30%, a normalised AGC target of 300%, a first mass of 110 Th and a max fill time of 25mS for a single microscan. All spectral data was collected in profile mode. Proteomics data are available via ProteomeXchange with identifier PXD047606. Protein matches, Peptide Modifications and PTMs are also found in Additional File S1.

7.3 Lipid Analysis

Samples were diluted in MTBE (1:100) and centrifuged at 20,000xg for 3 minutes. The top 50µL supernatant was transferred to a glass autosampler vial with 300µL insert and capped. Quality control samples were made by pooling 10µL from each sample. LC-MS/MS analysis was performed on the samples using an Ultimate 3000 HPLC system (Thermo-Fisher) consisting of an HPG-3400RS high-pressure gradient pump, TCC 3000SD column compartment, and WPS 3000 autosampler and coupled to a 6600 TripleTOF Q-TOF mass spectrometer (SCIEX) with TurboV ion source. The system was controlled by SCIEX Analyst 1.7.1, DCMS Link and Chromeleon Xpress software. A 5µL sample volume was initially injected by pulled loop with a 150µL post-injection needle wash with 1:1 acetonitrile and isopropanol. Separations were performed using an Accucore C18 column (Thermo) with dimensions of 150mm length, 2.1mm diameter, and 2.6µm particle size, and with gradient chromatography, where mobile phase A, was water with 10mM ammonium formate and 0.1% formic acid and mobile phase B of 10:9:1 acetonitrile, isopropanol and water with 10mM ammonium formate with 0.1% formic acid. The mass spectrometer was ran in both positive and negative modes at 5500V in positive mode and -4500V in negative mode at 400°C, and data was acquired in an information-dependent manner. Isotopes within 4Da were excluded from the scan. Lipid matches are found in Additional File S2.

7.4 Metabolomic Analysis

A similar protocol to lipidomics was carried out with a few modifications. Samples were resuspended in acetonitrile/water at a ratio of 5:1. The post-injection needle was washed with 9:1 acetonitrile and water, with separations on a Poroshell 120 HILIC-Z column (Agilent) with column dimensions of 150mm length, 2.1mm diameter and 2.7µm particle size equipped with a guard column of the same phase. Mobile phase A, was water with 10mM ammonium acetate, ammonium hydroxide and 20µM medronic acid, and Mobile phase B, was 85:15 acetonitrile and water with 10mM ammonium acetate, both phases adjusted to pH9 with ammonium hydroxide and 20µM medronic acid. The mass spectrometer was run in negative mode at -4500V, and data was acquired in a data-independent manner, using SWATH in the range of 50–1000 m/z, split across 78 variable-size windows (**Table S2**), each with an accumulation time of 20mS and a total cycle time of 1.66 seconds. Metabolite matches are found in Additional File S3.

8. Data analysis

8.1 Genomics

8.1.1 DNA sequences processing and mapping

Demultiplexing of raw data was performed using Illumina's bcl2fastq software which converts the raw BCL data output into fastq files. Residual adapter sequences were removed using AdapterRemoval v2.1[13], and the reads were trimmed to a minimum length of 28 nucleotides. Reads that had an overlap of 11bp were collapsed into single sequences. Trimmed and filtered sequences were mapped to the *Sus Scrofa* reference genome assembly, *Sscrofa11.1* (GenBank assembly GCA_000003025.6) using the Burrows-Wheeler Aligner (BWA) v.0.7.5a-r405[14] with the *aln* mapping algorithm and disabling seeding (*-l 1024).* SAM files were produced using BWA's *samse* command for collapsed reads. Resulting SAM files were cleaned and sorted by coordinate and converted to BAM format using default *CleanSam* and *SortSam* commands in PicardTools v2.18.27(http://broadinstitute.github.io/picard/). Mapped reads were extracted using Samtools v1.2 with parameters *b*, *q25* and *F4*. Duplicate sequences were removed using the aweSAM collapser script (https://gist.github.com/jakeenk/). Following duplicate removal, the collapsed reads were visualised on Geneious Prime 2023.1.2 (https://www.geneious.com). Quality Control was performed using FastQC (https://www.bioinformatics.babraham.ac.uk/projects/fastqc/). BLASTn searches were performed with somewhat similar sequences in four genes involved in brain function.

8.1.2 Degradation estimates

Bayesian estimation of degradation could not be computed with mapDamage[15] as there was not a significant amount of damage present in the sequences. We followed a qualitative approach including FastQC to assess the sequence read length, while fragmentation and base quality values were obtained through on Geneious Prime 2023.1.2 (https://www.geneious.com).

8.1.3 Single Nucleotide Polymorphism (SNP) calling

Prior to SNP calling a *Base Quality Score Recalibration* (BQSR) was performed according to GATK's[16] best practices, to account for errors in over- and under-estimated base quality scores in the data and improve the accuracy of variant calling. Single Nucleotide Polymorphism (SNP) calling was performed using GATK's[16] best practices with a few alterations. For this step, we used the archived *Sus Scrofa* SNP database from the National Centre for Biotechnology Information (NCBI). The *HaplotypeCaller, SelectVariants* and *Variant filtration* walkers were used with default parameters apart from RMSMapping Quality filter (MQ < 30). An additional parameter of Depth of Coverage was also used (DP10 for mitochondrial SNP calling and DP5 for nuclear SNP calling). Variants were recovered for all chromosomes and the mitochondrial genome.

8.2 Proteomics

Raw spectral data files were identified and scored using MaxQuant[17] 2.0.1.0. Searches were performed against Uniprot's pig proteome (UP000008227) using the following parameters: precursor tolerance of 10ppm, and main mass tolerance of 4.5ppm; tolerances for fragment ions of 0.5 Da; trypsin specificity with a maximum of two missed cleavages; carbamidomethylation was a fixed modification, and oxidation (M), acetylation (Protein N-term) and deamidation (NQ) were set as variable modifications; the required False Discovery Rates for both PSMs and peptides was set at 0.01 with a minimum required peptide length of six and a minimum score of 40 for modified proteins. PSMs from the MaxQuant[17] search were further subjected to statistical comparisons between different protocols using MSStats 4.6.3[18] in a RStudio 4.2.2 environment. The general MaxQuant[11] DDA analytical workflow was followed with the following parameters: for dataProcess, normalisation was set to "equaliseMedians" and summarisation to Tukey's median polish ("TMP"). Systematic biases between runs were evaluated using the quality control plot function (**Figure S7**). Featured proteins post-summarisation were tallied for the total number per sample and categorised into five general classes related to brain-specific biological functions (**Figures 1a-b**). Summarised and statistically significant peptides were then used to calculate total modifications and percentage deamidation for both protocol and timepoint. Peptides with multiple deamidation sites identified were only counted once to reduce search bias (Additional File S1). All protein modifications were searched

against UniProt to confirm whether identified deamidation sites are in vivo post-translational modifications (PTMs), of which none were identified. The group comparison function then calculated the fold changes of differential protein abundances between protocols for each sample (**Figure S8**). Heatmap figures, pie charts, and bar charts were also created in Rstudio.

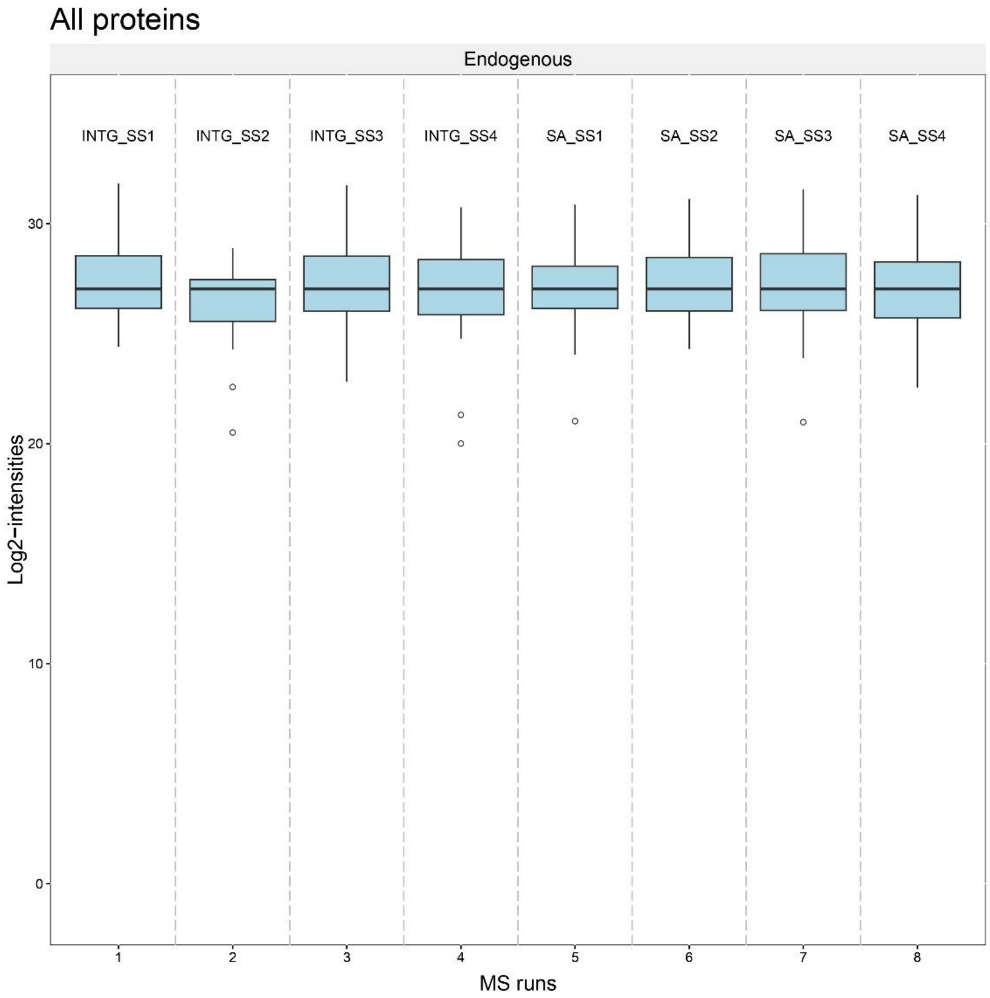

Figure S7: Proteomics Quality Control between each MS run.

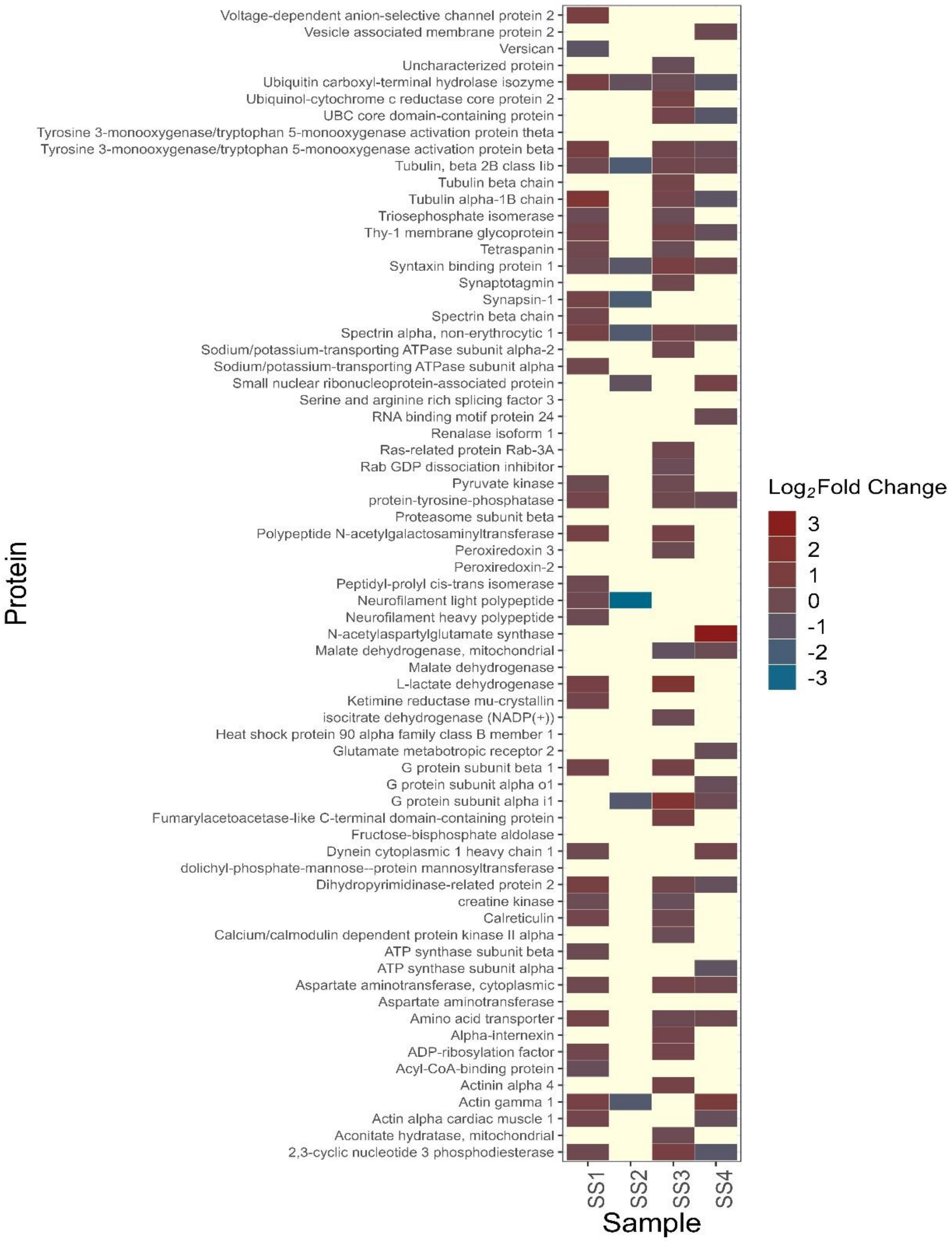

Figure S8: Heat Map of Individual Protein Abundance: Contrast matrices were used to compare the fold change in differential abundance across individual proteins between the same samples of each protocol and statistically validated; of the 117 proteins compared, 61 had positive fold change towards INTG, with 57 of these being statistically significant. In contrast, the nine largest fold changes in favour of the standalone protocol (out of 52 values) were between SS2.

8.3 Lipidomics and Metabolomics

MS and MS/MS peaks for both lipids and metabolites were identifications using MS-DIAL (ver. 4.9.22)[19]. Raw .wiff and .wiffscan files for lipids in both positive and negative modes were matched to the internal LipidBlast database (version 68), containing 81 classes, 377,313 molecules and 554,041 spectra in the Positive Library and 94 classes, 356,477 molecules, and 792,757 spectra in the Negative Library. The following analysis parameters used: a minimum 1000 peak height, 0.1Da slice width and a 0.5 sigma window value; retention tolerance was 100 minutes, MS1 tolerance was set to 0.01Da and MS2 tolerance to 0.05Da, with an ID score cutoff at 80%. Adducts for [M+H]+, [M+H-H2O]+ and [M+H-2H2O]+ for Positive Mode and [M+H]-, [M+H-H2O]- for Negative Mode were chosen in a CH3COONH4 solvent. Lipid family analysis was carried out by selecting peak IDs matched with Ref. matched, Suggested and with MS2 acquired filters for further processing in RStudio. All RIKEN artefact matches comparative to negative controls and MS blanks were removed, and all lipid classes were grouped within their main families as per nomenclature[20], with only fatty amides and fatty acyls separated to distinguish oxo fatty acid chains without a head group. A similar workflow was used to compare lipid classes, except only peaks filtered with Ref. matched and MS2 acquired were chosen.

Metabolites were also analysed using the combined MS-DIAL/Rstudio workflow, and required the selection of a suitable database. After comparing databases from MassBank, HMDB, MetaboseBASE, and LipidBlast, MS-DIAL's ESI(-)-MS/MS authentic standards database (MSMS_Public_EXP_NEG_VS17) was chosen due to the largest range of 9033 unique compounds and 4439 records that could be identified and the manageable size of MSP file for multiple computational runs. The SWATH-MS method was chosen to match data acquisition and include the 79 scan windows (**Table S3**), and the following analysis parameters were the same as Lipid Negative Mode. For data processing, selecting peak IDs matched with Ref. matched, Suggested and with MS2 acquired filters were used, with artefacts containing RIKEN, NCG, Pesticide and stereoisomeric compounds found within negative controls and MS blanks removed. Statistical analysis for PeakID datasets was performed in Graphpad Prism 9.4.1 to compare abundance of recovered and peak-matched small molecules (**Figure S9**).

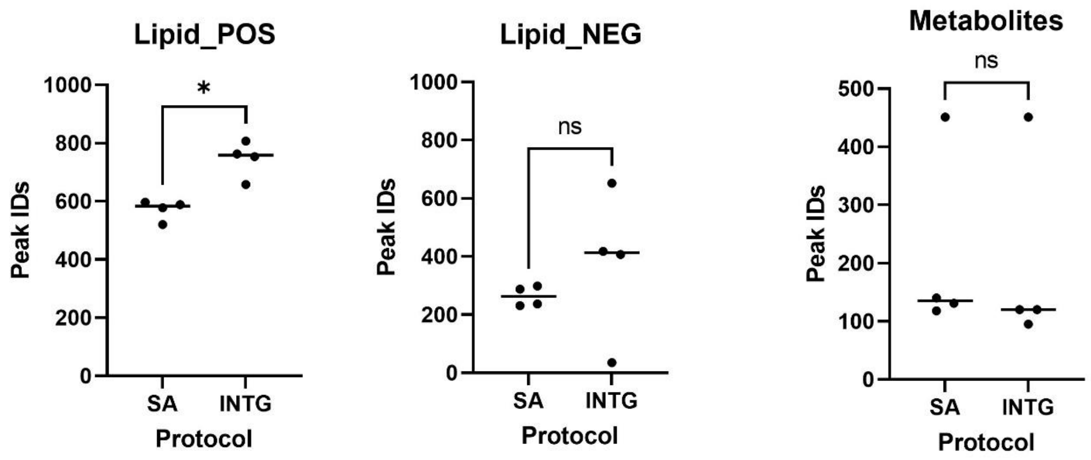

Figure S9: A Mann-Whitney Unpaired Test between Integrated and Standalone protocols for Lipidomics and Metabolomics Peak IDs.

REFERENCES

1. da Silva, B. P. *et al.* Brain fatty acid and transcriptome profiles of pig fed diets with different levels of soybean oil. *BMC Genomics* **24**, 91 (2023).
2. Miles, K. L., Finaughty, D. A. & Gibbon, V. E. A review of experimental design in forensic taphonomy: moving towards forensic realism. *Forensic Sciences Research* **5**, 249–259 (2020).
3. Matuszewski, S. *et al.* Pigs vs people: the use of pigs as analogues for humans in forensic entomology and taphonomy research. *Int J Legal Med* **134**, 793–810 (2020).
4. Larkin, B., Iaschi, S., Dadour, I. & Tay, G. K. Using accumulated degree-days to estimate postmortem interval from the DNA yield of porcine skeletal muscle. *Forensic Sci Med Pathol* **6**, 83–92 (2010).
5. Bernevic, B. *et al.* Degradation and oxidation postmortem of myofibrillar proteins in porcine skeleton muscle revealed by high resolution mass spectrometric proteome analysis. *International Journal of Mass Spectrometry* **305**, 217–227 (2011).
6. Dawidowska, J., Krzyżanowska, M., Markuszewski, M. J. & Kaliszan, M. The Application of Metabolomics in Forensic Science with Focus on Forensic Toxicology and Time-of-Death Estimation. *Metabolites* **11**, 801 (2021).
7. Poinar, H. N. The top 10 list: criteria of authenticity for DNA from ancient and forensic samples. *International Congress Series* **1239**, 575–579 (2003).
8. Dabney, J. *et al.* Complete mitochondrial genome sequence of a Middle Pleistocene cave bear reconstructed from ultrashort DNA fragments. *Proceedings of the National Academy of Sciences* **110**, 15758–15763 (2013).

9. Allentoft, M. E. *et al.* Population genomics of Bronze Age Eurasia. *Nature* **522**, 167–172 (2015).
10. Bligh, E. G. & Dyer, W. J. A rapid method of total lipid extraction and purification. *Can. J. Biochem. Physiol.* **37**, 911–917 (1959).
11. Meyer, M. & Kircher, M. Illumina sequencing library preparation for highly multiplexed target capture and sequencing. *Cold Spring Harb Protoc* **2010**, pdb.prot5448 (2010).
12. Kircher, M., Sawyer, S. & Meyer, M. Double indexing overcomes inaccuracies in multiplex sequencing on the Illumina platform. *Nucleic Acids Research* **40**, e3 (2012).
13. Schubert, M., Lindgreen, S. & Orlando, L. AdapterRemoval v2: rapid adapter trimming, identification, and read merging. *BMC Research Notes* **9**, 88 (2016).
14. Li, H. & Durbin, R. Fast and accurate short read alignment with Burrows–Wheeler transform. *Bioinformatics* **25**, 1754–1760 (2009).
15. Jónsson, H., Ginolhac, A., Schubert, M., Johnson, P. L. F. & Orlando, L. mapDamage2.0: fast approximate Bayesian estimates of ancient DNA damage parameters. *Bioinformatics* **29**, 1682–1684 (2013).
16. McKenna, A. *et al.* The Genome Analysis Toolkit: A MapReduce framework for analyzing next-generation DNA sequencing data. *Genome Res.* **20**, 1297–1303 (2010).
17. Cox, J. & Mann, M. MaxQuant enables high peptide identification rates, individualized p.p.b.-range mass accuracies and proteome-wide protein quantification. *Nat Biotechnol* **26**, 1367–1372 (2008).

18. Choi, M. *et al.* MSstats: an R package for statistical analysis of quantitative mass spectrometry-based proteomic experiments. *Bioinformatics* **30**, 2524–2526 (2014).
19. Tsugawa, H. *et al.* MS-DIAL: data-independent MS/MS deconvolution for comprehensive metabolome analysis. *Nat Methods* **12**, 523–526 (2015).
20. Liebisch, G. *et al.* Update on LIPID MAPS classification, nomenclature, and shorthand notation for MS-derived lipid structures. *Journal of Lipid Research* **61**, 1539–1555 (2020).

Table S1: Samples used in this study.

Sample ID	Tissue Type	Sample Weight (mg)
SS1	Cerebral Cortex	30
SS2	Cerebral Cortex	30
SS3	Cerebral Cortex	30
SS4	Cerebral Cortex	30

Table S2: Desiccation conditions per sample and observations.

Sample ID	Mass Before (grams)	Mass After (grams)	Observations	Av. Temperature (°C)	Av. Humidity (%)	Mass Loss %
SS1	27.7	11.06	Tissue soft inside, hard outside, smell of putrefaction	39.3	11	9.93
SS2	39.92	16.16	Solid tissue throughout, strong smell of putrefaction	39.1	12	40.48
SS3	33.88	11.92	Tissue softer and rubbery inside, solid outside, smell of putrefaction	39.5	12	35.18
SS4	34.95	8.72	Outside tissue hard, hollow and smells like putrefaction. No wet tissue	39.8	12	24.95

Table S3. m/z ranges for each variable SWATH window in data acquisition and analysis

Start m/z	End m/z
45	1000
49.5	76.3
75.3	82.8
81.8	87
86	91
89.7	94.7
93	98
96.2	101.2
100.2	105.2
104.2	109.2
108.2	113.2
112.2	117.2
116.2	121.2
120.2	125.2
124.2	129.2
128.2	133.2
132.2	137.2
136.2	141.2
140.2	145.2
144.2	149.2
148.2	153.2
152.2	157.2
156.2	161.2
160.2	165.2
164.2	169.2
168.2	173.2
172.2	177.2
176.2	181.2
180.2	185.2
184.2	189.2
188.2	193.2
192.2	197.2
196.2	201.2
200.2	205.2
204.2	209.2
208.2	213.2
211.3	216.3
215.1	220.7
219.7	225.8

224.8	231
230	236.6
235.6	243.1
242.1	249.6
248.6	256.1
255.1	264.1
263.1	272
271	279.4
278.4	288.3
287.3	297.1
296.1	306.5
305.5	315.3
314.3	323.7
322.7	332.6
331.6	340.9
339.9	349.8
348.8	358.7
357.7	367.5
366.5	376.4
375.4	387.1
386.1	399.2
398.2	412.7
411.7	425.8
424.8	445.8
444.8	469.1
468.1	496.1
495.1	529.2
528.2	559.5
558.5	587.5
586.5	628.5
627.5	694.2
693.2	760.3
759.3	879.6
878.6	977.5
976.5	981.5
980.5	985.5
984.5	989.5
988.5	993.5
992.5	997.5
996.5	1001.5
